# Genicular Artery Embolization for Chronic Exudative Knee Synovitis: Prospective 6-Month Clinical and Functional Outcomes

**DOI:** 10.3390/jcm15135172

**Published:** 2026-07-02

**Authors:** Małgorzata Drelich, Maciej Szmygin, Magdalena Sobiech, Paweł Kuczyński, Izabela Świetlicka, Karolina Turżańska, Sławomir Zaborek, Maryla Kuczyńska, Michał Sojka, Jacek Gągała, Silvija Ille, Tomasz Blicharski

**Affiliations:** 1 Department of Rehabilitation, Medical University of Lublin, 20-059 Lublin, Poland; 2 Department of Interventional Radiology and Neuroradiology, Medical University of Lublin, 20-059 Lublin, Poland; 3 Department of Sports Medicine, Medical University of Lublin, 20-059 Lublin, Poland; 4 Department of Biophysics, Faculty of Environmental Biology, University of Life Sciences in Lublin, 20-950 Lublin, Poland; izabela.swietlicka@up.edu.pl; 5 Department of Rehabilitation, University Clinical Hospital No. 4 in Lublin, 20-090 Lublin, Poland; 6 Department of Vascular Surgery, Medical University of Lublin, 20-059 Lublin, Poland; 7 Department of Orthopedics and Traumatology, Medical University of Lublin, 20-059 Lublin, Poland; 8 Oxford University Hospitals NHS Foundation Trust, Oxford OX39DU, UK

**Keywords:** genicular artery embolization, exudative synovitis, osteoarthritis, muscle strength, range of motion

## Abstract

**Background/Objectives**: The aim of this study is to analyze outcomes in patients who underwent genicular artery embolization (GAE) with respect to pain, symptoms, physical activity, quality of life, and knee joint range of motion and muscle strength of the knee joint. The study will provide evidence of the treatment’s effectiveness using both subjective and objective measurements. In addition, the study will examine the impact of the severity of radiological changes in the knee joint and the number of embolized vessels on the extent of improvement in outcomes following the procedure. **Methods**: Patients eligible for GAE who exhibited symptoms of chronic exudative knee arthritis, confirmed by USG and MRI with contrast, did not have laboratory markers of inflammation, nor did they respond effectively to standard non-surgical treatments. The analysis included 34 patients. Subjective parameters were assessed using the VAS and KOOS scales. Knee range of motion and muscle strength were assessed using an electronic dynamometer and goniometer. Measurements were performed according to the same schedule before GAE, and at 1, 3 and 6 months after the procedure. The results were subjected to statistical analysis. **Results**: In the analysis up to 6 months, a significant time effect was demonstrated for the VAS and all KOOS subscales. The improvement was visible after 1 month and continued after 3 and 6 months. No significant changes over time were demonstrated for objective parameters. Exploratory analysis demonstrated a relationship between increased knee extensor strength and pain reduction up to 3 months and increased range of motion up to 6 months after the GAE. There was no significant association between radiological changes and the number of embolized vessels with improved outcomes after GAE. **Conclusions**: GAE appears to be a promising minimally invasive treatment option for patients with chronic knee synovitis. Clinically meaningful improvements were observed in patient-reported outcomes.

## 1. Introduction

The knee joint is one of the largest joints in the human body and one of the most susceptible to injury and damage. When the synovial membrane of the knee joint becomes excessively irritated due to injury, overuse, or a condition such as osteoarthritis or rheumatoid arthritis, exudative synovitis develops. It manifests as pain of varying intensity, leading to reduced mobility. Chronic inflammation with recurrent effusions can contribute to synovial hypertrophy, accompanied by pathological neogenesis and neoinnervation, as well as the destruction of joint surfaces [1,2,3]. This results in reduced joint mobility, difficulty bearing weight on the limb, and difficulty moving, leading to muscle weakness and atrophy, as well as functional limitations, thereby affecting the patient’s quality of life. Most often, these changes are associated with the progression of osteoarthritis (OA).

Many clinical practice guidelines help physicians treat knee osteoarthritis and synovitis [4]. The treatment approach is based on a stepwise model ranging from physical therapy to pharmacological treatment and interventional procedures, with joint replacement serving as the final stage [5]. Higher-quality guidelines recommend implementing exercise, education, and weight loss as first-line treatment. It is also recommended to consider nonsteroidal anti-inflammatory drugs, taking into account any comorbidities [4].

Recommendations regarding intra-articular injections vary [4,6]. Glucocorticosteroid (GCS) injections effectively reduce pain in the short term (up to 6 weeks) but do not provide lasting clinical benefit [7,8]. A twofold higher risk of structural progression on imaging was also demonstrated in patients receiving intra-articular glucocorticoids [9]. Scientific evidence suggests that hyaluronic acid (HA) injections may provide moderate improvement in pain and function, but the effect usually lasts 3–6 months, and up to 12 months in some cases [10]. The effect of HA on the course of the disease has not been conclusively confirmed in clinical trials. Platelet-rich plasma (PRP) is an autologous blood-derived preparation containing numerous biologically active factors: high concentrations of platelets, growth factors, and cytokines. These factors are believed to modulate inflammatory processes and promote tissue regeneration, but the level of evidence is low [11]. Studies have confirmed the effectiveness of PRP in relieving pain and improving joint function [12]. PRP effects manifest gradually but last longer than six months. Combining therapy HA with PRP or corticosteroids offers short-term relief and long-term joint protection through synergistic effects [10]. Bone marrow aspirate concentrate (BMAC) is also used in biological therapy; it contains mesenchymal stromal cells, progenitor cells, and platelets, as well as numerous cytokines and growth factors [13]. Variations in preparation methods, injection protocols, and eligibility criteria make the practical application of this method difficult [14]. A comparison of the efficacy of injections, including GCS, HA, PRP, and BMAC, showed that HA and PRP, compared to placebo, resulted in a significant improvement in pain perception, and that HA, PRP, and BMAC led to a significant functional improvement compared to placebo over a follow-up period of at least 6 months [15]. Intra-articular injections provide immediate pain relief, and physical therapy plays a key role in maintaining joint function, especially in long-term treatment. Combining these two methods can yield comprehensive benefits [16]. For patients in whom conventional non-surgical treatment is ineffective, the disease stage does not yet warrant joint replacement surgery, or who do not wish to undergo surgery, other treatment options are explored. These include radiofrequency ablation (RFA) and knee artery embolization (GAE).

RFA is a minimally invasive pain treatment method that involves selectively damaging the nerve fibers that transmit pain signals using high-frequency currents. It works by generating an electromagnetic field around the device’s tip, which transfers thermal energy to the surrounding tissues [17]. RFA of the genicular nerve results in pain relief [18,19]. The results of randomized trials indicate that radiofrequency ablation (RFA) of the genicular nerves is superior to GCSs, HA injections, and oral analgesics [20]. At present, there is no convincing evidence that RFA directly treats synovitis, but the clinical results are promising.

Embolization is a minimally invasive procedure performed to reduce or eliminate abnormal blood vessel growth. It involves the percutaneous insertion of a vascular catheter and the occlusion of the vessel using an embolization material. It does not require general anesthesia and allows for a quick return home and a return to daily activities. It is believed that by reducing blood flow through the synovial membrane, GAE modulates inflammation, angiogenesis, and nociceptive signaling while maintaining overall blood supply to the joints [21]. This method has not yet been included in the major international guidelines on osteoarthritis. However, it is positioned as an adjunctive or bridging procedure between conservative treatment, injection therapy, and surgical treatment. The eligibility criteria for GAE are constantly evolving. Many authors consider GAE to be more biologically sound than RFA because it reduces inflammation and is less invasive than arthroscopic synovectomy, which mechanically removes the hypertrophied synovial membrane. A synovectomy is most commonly performed for inflammatory diseases to reduce pain, but it does not prevent disease recurrence or lesion progression [22]. There are no head-to-head studies comparing GAE with RFA or synovectomy. In their review, Sajan et al. compared pain reduction outcomes on the VAS in patients who underwent intra-articular injections, RFA, and GAE, and found no significant differences [23].

This was the first time GAE was performed to treat recurrent bleeding into the knee joint following total knee arthroplasty [24]. A review study by Melian et al. confirms improvement in 72.6% of cases involving intra-articular bleeding [25]. The results of embolization for the treatment of mild to moderate osteoarthritis were first reported in 2015 by Okuno et al. [26]. Currently, an increasing number of centers are performing these procedures while conducting research on their effectiveness, including comparisons with placebo (sham treatments) and other standard therapies (studies GENESIS and MOTION) [27,28,29,30]. Scientific reports indicate that this treatment method is effective in reducing pain and improving the patient’s functional status. The study also showed a 27% reduction in the number of OA patients using opioid medications and a 65% reduction in the use of nonsteroidal anti-inflammatory drugs (NSAIDs) following GAE treatment, while the number of intra-articular hyaluronic acid injections decreased by 73% [31].

The first genicular artery embolization procedure in Poland was performed in September 2024 at the University Clinical Hospital No. 4 in Lublin on a patient with OA. The rapid positive outcome of the treatment motivated the design of a prospective study to evaluate the efficacy of GAE in patients with symptoms of chronic synovitis, both subjectively and objectively. Published studies, reviews, and meta-analyses provide us with insights into patient selection, surgical techniques, embolization agents, and subjective outcomes [32,33,34,35,36,37]. Radiological examinations confirm a reduction in pathological vascularization, inflammation, and synovial hypertrophy of the knee joint, without affecting structural changes [38,39].

The aim of this study is to analyze the outcomes in patients who underwent embolization of the synovial vessels of the knee joint with regard to pain, symptoms, physical activity, and quality of life, as well as the range of motion and muscle strength of the knee joint. The study will provide evidence of GAE’s effectiveness and allow us to assess whether the patient’s reported improvement is reflected in objective measurements. In addition, the study will examine the impact of the severity of radiological changes in the knee joint and the number of embolized vessels on the extent of improvement in outcomes following the procedure.

## 2. Materials and Methods

### 2.1. Study Design

A prospective study of patients with symptoms of chronic exudative knee arthritis undergoing genicular artery embolization to eliminate pathological synovial vessels has been underway at University Clinical Hospital No. 4 in Lublin since January 2025 and is still ongoing. This study was approved by the Bioethics Committee of the Medical University of Lublin (No. KB-0024/7/01/2025, dated 23 January 2025). Participation in this study is voluntary.

Between January 2025 and 30 April 2026, the study included 36 patients who underwent genicular artery embolization, including 19 women and 17 men aged 37–78 (mean age: 59.42). GAE of a single knee was performed on 32 patients, and on both knees in 4 patients. The total number of knees treated was 40.

All participants to date were provided with detailed information about the study’s purpose, methodology, scope, and procedures, and they gave their informed written consent to participate in the study prior to its commencement.

### 2.2. Embolization Procedure

Patients with chronic synovitis of the knee joint resulting from osteoarthritis or rheumatoid arthritis with recurrent effusions were eligible for genicular artery embolization if standard treatments—including medication, physical therapy, and intra-articular injections—had failed to produce satisfactory results. Laboratory tests revealed no elevated inflammatory markers (leukocytosis, C-reactive protein). Diagnostic imaging studies (contrast-enhanced ultrasound and contrast-enhanced magnetic resonance imaging of the knee joint) confirmed the presence of abnormal vessels in the synovial membrane.

All genicular artery embolization (GAE) procedures were carried out by interventional radiologists with at least 6 years of experience in endovascular treatment. Under sterile conditions, an ultrasound-guided anterograde 4–5 Fr vascular access was obtained and initial angiography from the catheter placed in the distal superficial femoral artery was carried out to visualize the genicular arterial anatomy and confirm the presence of hyperemic blush. Target vessels supplying the pathological synovium were selectively catheterized with a microcatheter (size ranging from 1.7 to 2.0 Fr) and cautiously embolized with 100–300 μm particles (either resorbable or permanent) with the aim of “pruning” the abnormal vasculature and preserving native distal genicular branches. Technical success was defined as selective catheterization and embolization of the target genicular arteries. Control angiography was performed in order to evaluate the embolic effect and potential non-target embolization. The access site was compressed for at least 10 min, and a compression bandage was applied. The procedure lasted approximately 60 min. Patients were advised to keep the compression dressing in place for at least 4–6 h after the procedure and to remain lying down. Patients were discharged home the following day.

### 2.3. Study Inclusion Criteria

The inclusion criteria for the study were: consent to participate in the study and to the processing of personal data; age over 18; absence of acute inflammation; a minimum interval of 6 weeks since the last joint injection; a minimum interval of 3 months since the last physiotherapy treatment; radiographic degenerative changes graded 1–3 according to the Kellgren-Lawrence (KL) classification. The exclusion criteria were: refusal to participate in the study or to consent to the processing of personal data; acute inflammation of the knee joint confirmed by laboratory tests; skin lesions at the site where the measuring devices are applied; suspected malignant lesion of the knee joint; grade 4 radiographic degenerative changes according to the KL classification.

### 2.4. Patient Assessment

The initial assessment of patients was conducted the day before hospitalization or on the day of admission (study B1). Subsequent assessments were conducted 1 month after the procedure (study B2), 3 months after the procedure (study B3), 6 months after the procedure (study B4), and 12 months after the procedure (study B5). Each study employed a protocol of repeated assessments. At no stage of the studies did the patient receive additional recommendations regarding rehabilitation or pharmacotherapy.

To obtain basic information about the patients, a proprietary questionnaire was used to collect data on demographics (age, sex, height, and body weight for assessing Body Mass Index (BMI) and nutritional status), history of knee symptoms, underlying conditions and comorbidities, and current treatment (oral medication, intra-articular injections, physical therapy). The extent of radiographic changes was determined based on the Kellgren-Lawrence classification.

The Visual Analog Scale (VAS) was used to assess pain intensity. It took the form of a ruler with markings from 0 to 10, where 0 indicated no pain and 10 indicated unbearable pain—that is, the most severe pain the patient could imagine. Pain intensity was assessed on the day of the examination, the night before the assessment, and the average for the week and month preceding the assessment.

The KOOS (Knee Injury and Osteoarthritis Outcome Study) scale was also used to evaluate the results of the embolization procedure. It consists of five sections covering the assessment of symptoms, pain, activities of daily living (ADL), sports and recreational activities (ASR), and quality of life (QOL) [40].

The range of motion (ROM) and the strength of the extensor (EX) and flexor (FL) muscles of the knee joint on the treated and contralateral limbs were assessed using specialized electronic devices.

**Figure 1:**
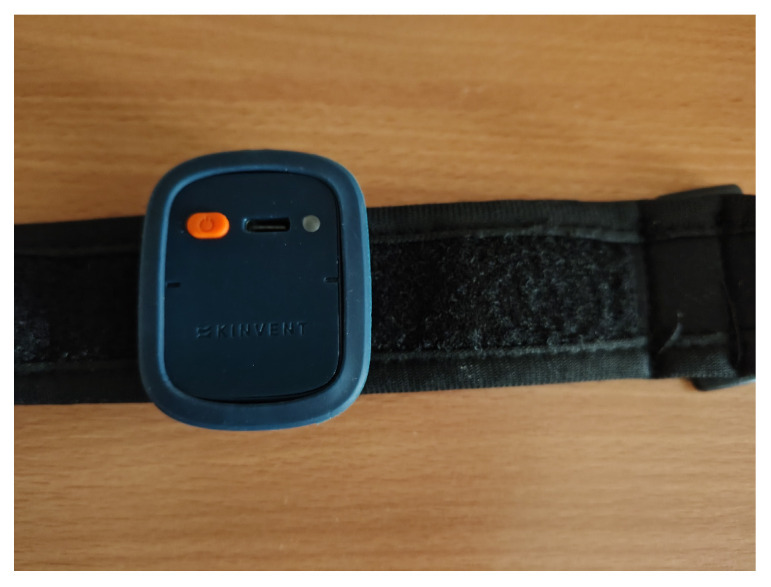
Electronic goniometer K-FORCE Move v3.

**Figure 2:**
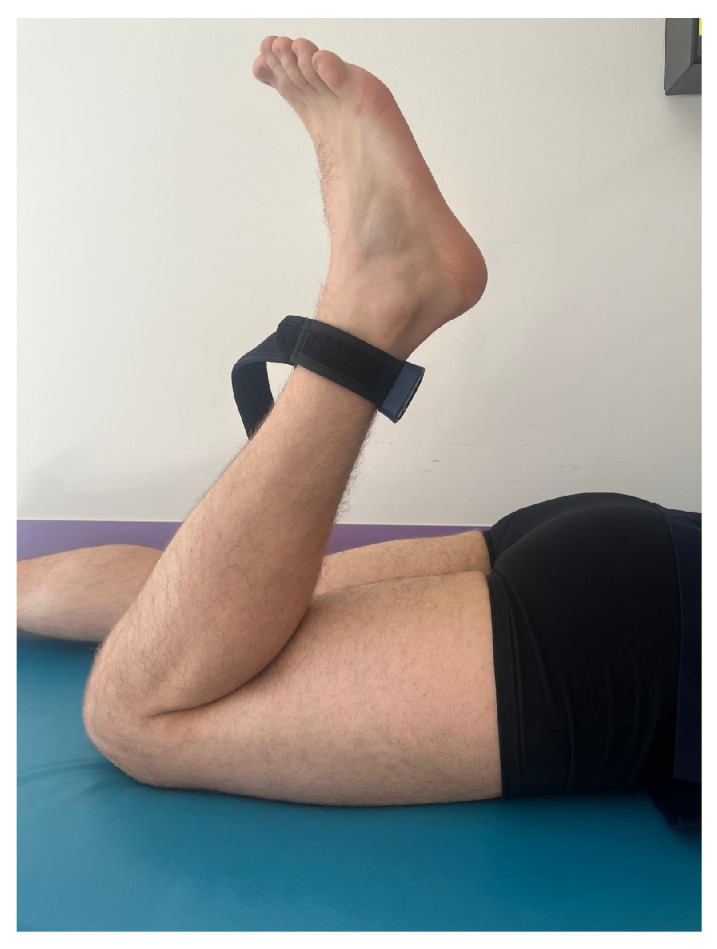
Knee flexion examination; the device’s sensor was placed on the distal part of the limb being examined, above the patient’s ankles, while the patient was lying face down.

An electronic goniometer (K-FORCE Move v3, Kinvent, Montpellier, France) was used to measure range of motion in accordance with the algorithm included in the software (Figure 1). The device’s sensor was placed on the distal part of the limb being examined, above the patient’s ankles, while the patient was lying face down. On command, the patient performed a movement involving maximum knee flexion (Figure 2), held that position for 3 s, and returned to the starting position. The procedure was repeated three times. The rest period after movement was 5 s, and the preparation time was 5 s. The average flexion angle [°] for each limb and the percentage of asymmetry [%] were recorded.

**Figure 3:**
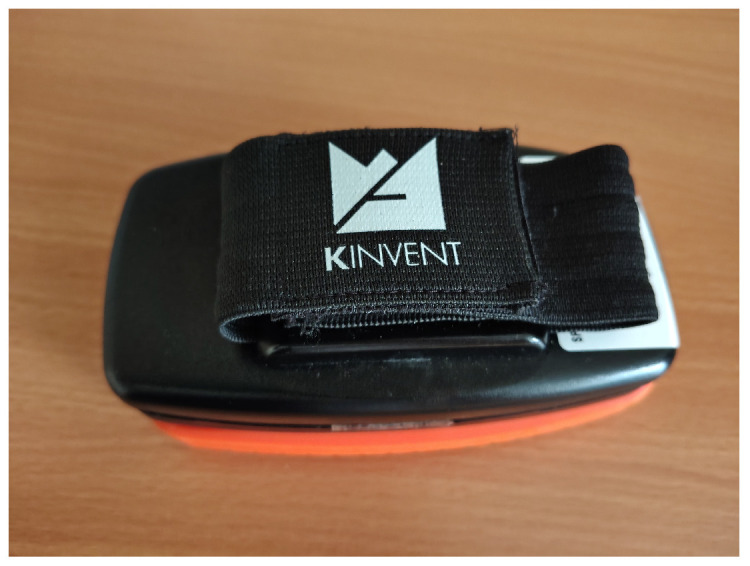
K-FORCE Muscle Controller handheld dynamometer.

**Figure 4:**
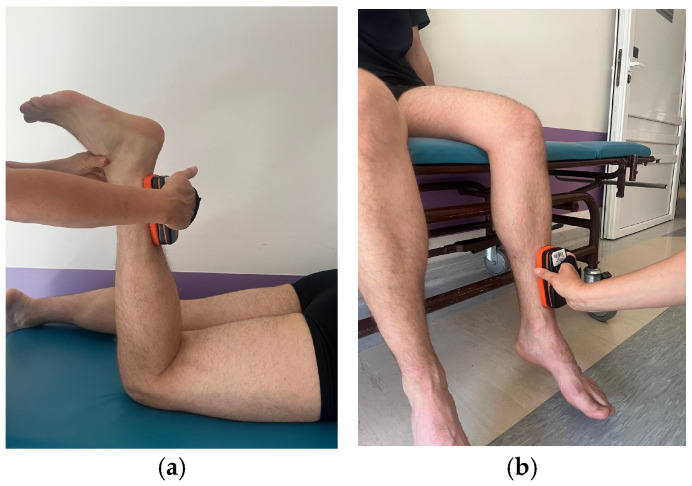
Muscle strength examination: (**a**) flexor muscle strength test in the prone position with the knee flexed at 90 degrees; the device was placed against the distal posterior aspect of the shin; (**b**) extensor muscles test in a seated position with the legs hanging off the bench; the device was placed on the anterior aspect of the distal lower leg.

A K-FORCE Muscle Controller handheld dynamometer (Kinvent, Montpellier, France) was used to measure knee joint muscle strength (Figure 3). The measurement of knee flexor muscle strength was performed in the prone position with the knee flexed at 90 degrees, and the measuring device was placed against the distal posterior aspect of the shin (Figure 4a). The strength of the knee extensor muscles was measured in a seated position with the legs hanging off the bench, and the device was placed on the anterior aspect of the distal lower leg (Figure 4b). Muscle strength was measured during isometric muscle contraction. When instructed, the patient applied pressure to the device for 3 s and rested for 5 s. The procedure was repeated 3 times. The preparation time was 5 s. The average strength score, measured in kilograms [kg], and the percentage of asymmetry [%] were recorded.

Radiographs of the knee joints were evaluated by two orthopedic surgeons using the Kellgren-Lawrence classification system to assess the severity of degenerative changes.

The number of vessels embolized was determined based on the surgical report.

### 2.5. Study Population and Data Completeness

**Scheme 1:**
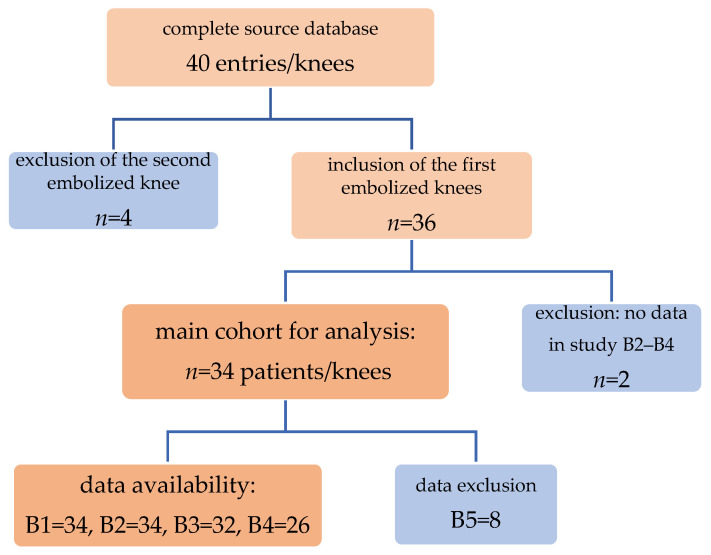
Patient flowchart with exclusion criteria. Study B1–B5: before the procedure (B1), one month after the procedure (B2), three months after the procedure (B3), six months after the procedure (B4), and 12 months after the procedure (B5).

The main cohort, comprising patients with a first episode of knee embolization and at least one post-procedure assessment, was included in the further analysis of the results. From the total database of 40 entries (knees), 4 cases involving the second knee in previously treated patients and 2 cases without useful post-procedure follow-up were excluded. Ultimately, the analysis included 34 patients and 34 knees that underwent embolization (Scheme 1).

The study employed a protocol of repeated assessments conducted prior to the procedure and at follow-up visits at 1, 3, 6, and 12 months (Table 1). In the main cohort, data were available for 34 participants at baseline (B1) and after 1 month (B2), 32 participants after 3 months (B3), and 26 participants after 6 months (B4). The 12-month study (B5) was excluded due to small datasets (8 participants). Given the very high data completeness through the third month, this time frame was considered the primary reference point for interpreting the results, while the six-month study was treated as an extension of the short-term observation.

### 2.6. Statistical Analysis

The descriptive statistics were calculated for the main cohort. Continuous variables are presented as the median and interquartile range [IQR], while categorical variables are presented as frequencies and percentages.

A time-series analysis was conducted for the main cohort, which included first episodes of knee embolization. The main analysis included measurements taken before the procedure and at 1, 3, and 6 months. Continuous variables are reported as median [IQR], and categorical variables as *n* (%). Changes over time were assessed using linear mixed models (LMMs) for repeated measures (Equation (1)). A separate model was fitted for each variable analyzed, including all available questionnaire scores (VAS, KOOS), objective variables regarding range of motion (ROM) and muscle strength (extensors, EX, and flexors, FL) on the treated side, as well as derived variables describing side asymmetry (difference in range of motion between the treated limb and the contralateral limb, ΔROM; the difference in extensor strength between the treated limb and the contralateral limb, ΔEX; the difference in flexor strength between the treated limb and the contralateral limb, ΔFL). In each model, time was treated as a categorical variable, and the patient ID as a random effect.

(1) $$Y_{ij}=\beta_{0}+\beta_{1}{assessment}_{ij}+u_{i}+\varepsilon_{ij}$$

where

*Y_ij_*—the value of the analyzed variable for the *i*-th patient in the *j*-th examination; *β*_0_—free term; *β*_1_*assessment_ij_*—time effect; *u_i_*—random patient effect; and *ε_ij_*—residual error.

A global time effect was reported, along with planned comparisons of results at 1, 3, and 6 months relative to baseline values. For contrasts, the *p*-value was calculated using the Holm correction for multiple comparisons within a given variable. The results tables present observed values as median [IQR], whereas model-based estimated differences from baseline, 95% confidence intervals (CIs), Holm-adjusted *p*-values, and standardized effect sizes were derived from these contrasts. Additionally, for each variable, the *p*-value for the overall time effect is provided. Standardized effect sizes were calculated by dividing the model-based contrast estimate by the residual standard deviation of the corresponding linear mixed model. Thus, medians [IQRs] describe the observed data, whereas estimated differences, CIs, *p*-values, and effect sizes are derived from the fitted models.

Because only two patients in the main cohort had rheumatoid arthritis (RA)-related synovitis, a formal subgroup analysis comparing osteoarthritis (OA)-related and RA-related synovitis was not statistically justified. To evaluate whether the inclusion of these two RA cases influenced the primary longitudinal findings, an additional diagnosis sensitivity analysis was performed. The primary linear mixed-model analysis was repeated after excluding the two RA cases using the same model structure, outcome variables, time points, planned comparisons with baseline, and Holm correction as in the main analysis. The results were compared with the primary analysis to assess the stability of the conclusions.

To assess whether the Kellgren-Lawrence (KL) grade of radiographic changes and the number of embolized vessels were associated with the extent of improvement following the procedure, additional exploratory analyses were conducted. The analyses were performed separately for changes from baseline to month 3 and separately for changes from baseline to month 6. The analyzed outcome was the change in the analyzed parameter relative to the baseline measurement, denoted by the letter d. For the VAS, the change was defined as the baseline value (B1) minus the value at a given follow-up point (B3 or B4), such that a positive value indicated a reduction in pain. For the KOOS subscales, range of motion (ROM), and muscle strength of the extensors (EX) and flexors (FL), the change was defined as the value at a given follow-up point (B3 or B4) minus the baseline value (B1), such that a positive value indicated improvement. For the parameters of side asymmetry (ΔROM, ΔEX, ΔFL), the change in the index value relative to the baseline measurement (B1) was analyzed, denoted as dΔROM, dΔEX, and dΔFL, respectively. The impact of the extent of radiological changes was assessed by comparing the magnitude of changes between patients with KL 2 and KL 3 using the Mann–Whitney U test. The relationship between the number of embolized vessels and the extent of improvement from baseline to 3 and 6 months was separately assessed using Spearman’s rank correlation. These analyses were supplementary and exploratory in nature. A *p*-value of <0.05 was considered statistically significant.

The relationships between subjective and objective changes were additionally assessed using Spearman’s correlation for changes (d) from baseline values (pre-treatment assessment) at 3 and 6 months. Correlations were assessed based on changes relative to the pre-procedure assessment, as the aim of this analysis was to determine whether greater subjective improvement after the procedure is accompanied by greater improvement in objective parameters; this approach minimizes the impact of baseline differences among patients and better addresses the question of the covariation of treatment effects.

An additional sensitivity power analysis was performed for objective functional parameters to clarify the magnitude of within-subject changes that could be detected with the available sample size. For each follow-up comparison with baseline, the minimum detectable standardized within-subject effect was estimated for 80% power and a two-sided alpha level of 0.05 using the available number of paired observations. For ROM, EX, and FL, the minimum detectable change in original units was calculated using the observed standard deviation of within-subject changes.

A robustness sensitivity analysis was conducted by excluding observations obtained following additional post-procedural interventions, such as rehabilitation, intra-articular injections, surgical treatment, or repeat embolization. The analysis was performed using the same linear mixed models as in the main analysis. The overall effect of time was assessed, along with planned comparisons relative to pre-procedure measurements at 1, 3, and 6 months, with a Holm correction for multiple comparisons. The results were compared with the main analysis to assess their stability. The aim of this analysis was to verify whether the main results remain stable after limiting the influence of subsequent co-interventions.

## 3. Results

### 3.1. Characteristics of the Study Group

The group of patients analyzed included 17 women (50.0%) and 17 men (50.0%). The median age was 60.0 years [52.2–63.8], and the median BMI was 32.0 kg/m^2^ [28.5–35.6]. In terms of body composition, 6 patients (17.6%) had a normal body weight, 7 (20.6%) were overweight, 11 (32.4%) had class I obesity, 8 (23.5%) had class II obesity, and 2 (5.9%) had class III obesity. The following comorbidities were identified: hypertension (HT) in 14 patients (14.2%), ischemic heart disease (IHD) in 3 (8.8%), atrial fibrillation (AF) in 2 (5.9%) and type 2 diabetes mellitus (T2DM) in 2 (5.9%) patients. All patients received physiotherapy, pharmacotherapy, and intra-articular injections as part of their treatment prior to the GAE procedure. None of the patients underwent arthroscopic surgery. Among the medications used, NSAIDs were the most common (34 patients; 100%), followed by opioids (4 patients; 11.8%), oral GCSs (3 patients; 8.8%), and methotrexate (MTX) (2 patients; 5.9%). The median number of intra-articular injections was 3 [3.0–4.0]. The most frequently administered intra-articular injections were HA (31 patients; 91.2%), PRP (22; 64.7%), and GCS (15; 44.1%) injections.

The median duration of symptoms prior to the procedure was 1.75 years [0.62–2.50]. The right knee underwent embolization in 18 patients (52.9%), and the left knee in 16 (47.1%). The most common diagnosis was osteoarthritis (OA) of the knee, which was found in 32 patients (94.1%), while two cases (5.9%) involved rheumatoid arthritis (RA). Grade 2 radiographic changes according to the Kellgren-Lawrence classification were found in 17 patients (50.0%), and Grade 3 in the remaining 17.

The average number of embolized vessels was 3 [3.0–4.0]. Immediate postoperative complications were reported in 2 patients (5.9%), and in both cases, it was an extensive hematoma. Long-term post-procedural rehabilitation was reported in 5 patients (14.7%), additional intra-articular injections in 3 (8.8%), and further surgical intervention also in 3 patients (8.8%) (surgery in 2 patients and repeat embolization in 1 patient) (Table 2). Oral corticosteroid therapy in patients with RA remained unchanged after the procedure. The use of analgesics in the remaining patients was as-needed and one-time.

### 3.2. Time-Series Analysis

**Figure 5:**
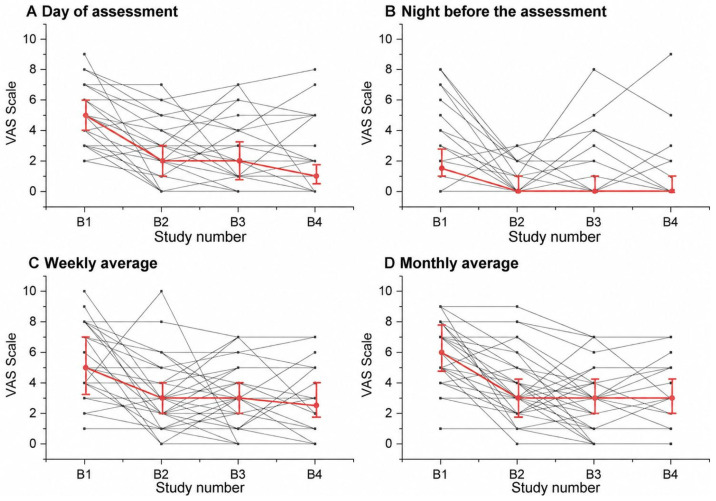
Changes in pain intensity assessed using the VAS at successive follow-up points after knee embolization. Individual patient profiles (gray lines) and the median with interquartile range [IQR] (red line) are presented for pain assessments: (**A**)—day of the assessment; (**B**)—night before the assessment; (**C**)—weekly average; (**D**)—monthly average. B1—baseline study; B2—1 month; B3—3 months; B4—6 months after the procedure.

**Figure 6:**
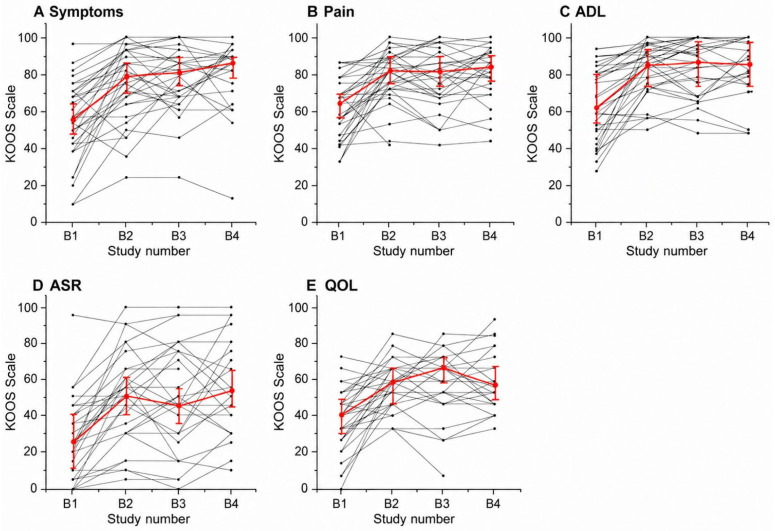
Changes in KOOS questionnaire scores at subsequent follow-up visits after knee embolization. Individual patient profiles (gray lines) and the median with interquartile range [IQR] (red line) are presented for the KOOS subscales: (**A**)—symptoms; (**B**)—pain; (**C**)—activities of daily living (ADL); (**D**)—sports and recreational activities (ASR); (**E**)—quality of life. B1—baseline study; B2—1 month; B3—3 months; B4—6 months post-procedure.

The analysis up to 6 months post-procedure demonstrated a significant time effect for all questionnaire scores, including all VAS and KOOS subscale scores (Table 3, Figure 5 and Figure 6). Model-based estimated changes from baseline, 95% confidence intervals, and standardized effect sizes for planned comparisons are presented in Table 3. Improvement was evident as early as 1 month post-procedure, persisted at 3 months, and remained present at 6 months. The median VAS score on the day of the study decreased from 5 [3–6] before the procedure to 2 [1–4] after 1 month, 2 [0–4] after 3 months, and 1.5 [0–4.5] after 6 months, while the KOOS pain subscale score increased from 64 [47–69] to 81 [72–88.25] after 1 month, 81 [68.5–89] after 3 months, and 83.5 [72.75–92] after 6 months. Significant improvements were also observed in the domains of symptoms, daily activities, sports and recreational activities, and quality of life. The standardized effect sizes confirmed large improvements in patient-reported outcomes, particularly for VAS pain reduction and KOOS subscales.

In contrast to the subjective findings, no significant changes over time were observed in the objective parameters of the embolized limb (Table 3). This applied to both the range of motion and the strength of the extensors and flexors. No significant changes were observed in the parameters describing asymmetry between the treated and contralateral limbs (ΔROM, ΔEX, ΔFL). This means that during the observed period of up to 6 months, patients reported clear clinical improvement, but it was not accompanied by an equally clear improvement in objectively measured functional parameters. The confidence intervals for objective outcomes were compatible with no relevant change as well as with small-to-moderate changes, supporting a cautious interpretation of these nonsignificant findings.

The sensitivity power analysis (Table 4) indicated that, given the available sample size, the study had 80% power to detect approximately moderate within-subject effects at 1 and 3 months and moderate-to-large effects at 6 months for objective functional parameters. In original units, the minimum detectable changes were approximately 7.12°, 7.63°, and 7.21° for ROM; 3.59 kg, 2.93 kg, and 2.81 kg for extensor strength; and 1.47 kg, 1.29 kg, and 1.38 kg for flexor strength at 1, 3, and 6 months, respectively. Therefore, smaller changes in ROM or muscle strength could not be reliably excluded.

### 3.3. Analysis of the Degree of Radiological Changes and the Number of Embolized Vessels on the Degree of Improvement Following the Procedure

The exploratory analysis included 32 cases with available data on changes from baseline to 3 months and 26 cases with available data on changes from baseline to 6 months. No significant differences in the degree of improvement were observed between patients with KL 2 and KL 3 at either 3 months or 6 months. This means that, in the study cohort, the severity of radiological changes did not significantly influence the changes in the assessed subjective and objective parameters following the procedure. The result closest to the significance threshold was for the change in nighttime pain after 3 months (dVAS—night before the examination, *p* = 0.058); however, statistical significance was not achieved in this case either (Table 5). Similarly, no significant correlations were found between the number of embolized vessels and the degree of improvement in most of the analyzed parameters at both 3 and 6 months (Table 6). The only exception was a single positive correlation between the number of embolized vessels and the change in extensor strength asymmetry at 3 months (dΔEX, rs = 0.364, *p* = 0.041).

In summary, no clear evidence was found in the study cohort that the degree of radiological changes as assessed by the Kellgren-Lawrence classification or the number of embolized vessels was significantly associated with the extent of clinical and functional improvement following the procedure. Given the exploratory nature of the analysis and the lack of confirmation of this association for other parameters and during the 6-month follow-up, this result should be interpreted with caution.

### 3.4. Correlations

**Figure 7:**
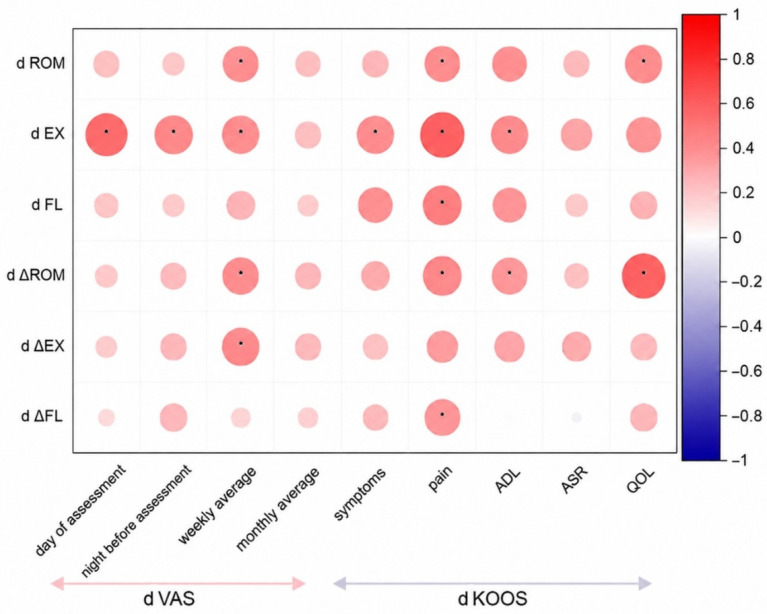
Spearman’s correlation map showing the relationship between changes in objective parameters and changes in questionnaire scores after 3 months (B3) relative to the baseline study (B1). The size of the circle corresponds to the absolute value of the correlation coefficient, and the color indicates the direction and strength of the relationship—from negative correlations (blue) to positive correlations (red). An asterisk indicates statistically significant relationships (*p* < 0.05). d denotes the change in a given parameter at 3 months relative to the pre-treatment assessment (B1). ROM—range of motion; EX—extensor strength; FL—flexor strength; ΔROM, ΔEX, and ΔFL—change in asymmetry between the treated limb and the contralateral limb in range of motion, extensor strength, and flexor strength, respectively; ADL—activities of daily living; ASR—sports and recreational activities; QOL—quality of life.

In an exploratory analysis of changes (d) from baseline to 3 months post-surgery, it was demonstrated that improvement in pain, as assessed by both the VAS and the KOOS-pain subscale, was primarily associated with an increase in the strength of the extensor muscles of the treated limb (Figure 7, Table 7).

Overall, greater reductions in pain were associated with greater increases in extensor strength (dEX). This pattern was observed for pain on the day of the assessment (rs = 0.497, *p* = 0.004), the night before the assessment (rs = 0.419, *p* = 0.017), and as the average pain over the week (rs = 0.402, *p* = 0.023). The same pattern was also seen for improvement in the KOOS-pain subscale (rs = 0.555, *p* < 0.001), as well as for improvement in symptoms (rs = 0.399, *p* = 0.024) and activities of daily living (rs = 0.411, *p* = 0.020). In the same study, improvement in pain was also associated with an increase in the range of motion of the treated limb (dROM). Significant positive correlations were found for a reduction in average weekly pain (rs = 0.370, *p* = 0.037) and improvement on the KOOS-pain subscale (rs = 0.367, *p* = 0.039). Additionally, a greater improvement in quality of life as assessed by the KOOS-QOL subscale was accompanied by a greater increase in range of motion (rs = 0.408, *p* = 0.020). With regard to flexor strength of the operated limb (dFL), a significant association was only demonstrated with improvement in the KOOS-pain subscale (rs = 0.430, *p* = 0.014), indicating that a greater reduction in pain was accompanied by a greater increase in flexor strength.

An analysis of changes in limb asymmetry showed that the reduction in pain and improvement in function were also accompanied by changes in the relationship between the treated and contralateral sides. For changes in range of motion asymmetry (dΔROM), significant positive correlations were observed with a reduction in average weekly pain (rs = 0.386, *p* = 0.029), improvement in KOOS-pain (rs = 0.427, *p* = 0.015), KOOS-ADL (rs = 0.372, *p* = 0.036), and KOOS-QOL (rs = 0.534, *p* = 0.002). For the change in extensor strength asymmetry (dΔEX), a significant association was only found with a reduction in average weekly pain (rs = 0.399, *p* = 0.024), while for the change in flexor strength asymmetry (dΔFL), a significant association was found with an improvement in KOOS-pain (rs = 0.369, *p* = 0.038).

In summary, after 3 months, improvement in pain was primarily associated with increased strength in the extensor muscles. It was followed by improvements in range of motion and a reduction in limb asymmetry.

**Figure 8:**
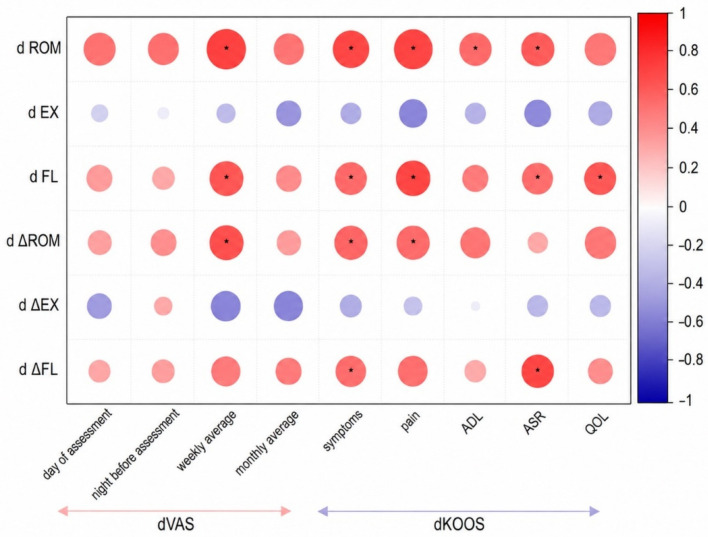
Spearman’s correlation map showing the relationship between changes in objective parameters and changes in questionnaire scores after 6 months (B4) relative to baseline measurements (B1). The size of the circle corresponds to the absolute value of the correlation coefficient, and the color indicates the direction and strength of the relationship—from negative correlations (blue) to positive correlations (red). An asterisk indicates statistically significant relationships (*p* < 0.05). d—the change in a given parameter at 6 months relative to the pre-treatment study (B1). ROM—range of motion; EX—extensor strength; FL—flexor strength; ΔROM, ΔEX, and ΔFL—change in asymmetry between the treated limb and the contralateral limb in range of motion, extensor strength, and flexor strength, respectively; ADL—activities of daily living; ASR—sports and recreational activities; QOL—quality of life.

In the analysis of changes from baseline to 6 months (Figure 8, Table 8), the most consistent pattern of association was observed for the range of motion of the treated limb (dROM). A greater reduction in pain based on the VAS (all *p* < 0.05) and a greater improvement in the KOOS subscales (all *p* < 0.05) were accompanied by a greater increase in range of motion. At 6 months, no significant correlations were found for changes in the strength of the extensor muscles of the treated limb (dEX). In contrast, changes in flexor strength (dFL) were found to be positively correlated with a reduction in average weekly pain (rs = 0.454, *p* = 0.020) and an improvement in the KOOS symptom (rs = 0.433, *p* = 0.027), pain (rs = 0.528, *p* = 0.006), sports and recreational activity (rs = 0.444, *p* = 0.023), and quality of life (rs = 0.483, *p* = 0.012) domains. Regarding changes in limb asymmetry, the largest number of significant correlations concerned changes in range of motion asymmetry (dΔROM). This parameter correlated positively with a reduction in average weekly pain (rs = 0.469, *p* = 0.016) and an improvement in KOOS scores for symptoms (rs = 0.429, *p* = 0.029), pain (rs = 0.489, *p* = 0.011), ADL (rs = 0.398, *p* = 0.044), and quality of life (rs = 0.411, *p* = 0.037). For the change in extensor strength asymmetry (dΔEX), the only significant negative correlation was observed with the change in average monthly pain based on the VAS (rs = −0.431, *p* = 0.028). In contrast, for the change in flexor strength asymmetry (dΔFL), significant positive correlations were observed with improvements in the KOOS symptom domain (rs = 0.401, *p* = 0.043) and in the sports and recreational activity domain (rs = 0.467, *p* = 0.016).

In summary, after 6 months, the subjective improvement was primarily associated with an increase in the range of motion of the treated limb. Subsequently, correlations were observed with increased flexor strength and changes in asymmetry between the limbs.

### 3.5. Sensitivity Analysis

An additional diagnosis sensitivity analysis was performed after excluding the two patients with rheumatoid arthritis (RA)-related synovitis. The OA-only cohort included 32 patients, with data available for 32 patients at baseline and 1 month, 30 patients at 3 months, and 24 patients at 6 months. The results were consistent with the primary analysis (Appendix A.1 Table A1). The VAS and all KOOS subscales retained a significant time effect, with significant improvements at 1, 3, and 6 months compared with baseline. In contrast, objective functional parameters showed the same pattern as in the primary analysis, with no significant global time effect for ROM, EX, FL, ΔROM, ΔEX, or ΔFL. Thus, excluding the two RA cases did not materially affect the main statistical conclusions.

In the second sensitivity analysis, after excluding observations obtained following additional post-procedural co-interventions, all questionnaire outcomes, including the VAS and KOOS subscales, retained a significant time effect (Appendix A.2 Table A2). Furthermore, planned comparisons with baseline at 1, 3, and 6 months remained significant, indicating that the observed improvement in pain, function, and quality of life was not materially affected by excluding observations potentially modified by subsequent co-interventions.

With regard to objective parameters, the picture was essentially similar to that obtained in the main analysis. For the range of motion, extensor strength, and flexor strength of the treated limb, as well as for parameters describing asymmetry between the limbs, no clear and consistent changes over time were observed. The only difference worth noting was that, for the treated limb’s range of motion, the comparison between the 3-month follow-up and the baseline examination approached the threshold of significance; however, the overall effect of time remained non-significant.

In summary, the sensitivity analysis confirmed the stability of the primary clinical outcome of embolization, characterized by marked improvements in pain, function, and quality of life, whereas no equally clear changes were observed in objectively measured parameters of limb function.

The important finding of this study was the discrepancy between patient-reported outcomes and objective patient measures. Patient reports presented a significant reduction in pain and improved quality of life, but this was not parallel by statistically significant improvements in range of motion or muscle strength. This finding suggest that separate pain reduction alone is insufficient to restore physical performance. Additional rehabilitation is needed to translate symptomatic improvement into measurable functional gains.

## 4. Discussion

In our study, the patient group consisted of 17 women (50.0%) and 17 men (50.0%). The median age was 60.0 years. The average duration of symptoms prior to genicular artery embolization was 1.75 years [0.62–2.50]. Grade 2 radiographic changes according to the Kellgren-Lawrence classification were observed in 17 patients (50.0%), and Grade 3 in the remaining 17. Patients eligible for embolization who exhibited symptoms of chronic exudative knee arthritis, confirmed by ultrasound examination with contrast and magnetic resonance imaging with contrast, did not have laboratory markers of inflammation, nor did they respond effectively to standard non-surgical treatments. In a group of 34 patients (34 knees), OA was the underlying cause of symptoms in 94% of cases. In 2 patients treated for RA and taking disease-modifying antirheumatic drugs, imaging studies of the knee joint revealed typical degenerative changes in the form of thinning of the articular cartilage and changes characteristic of chondropathy, with the presence of osteophytes on the bony margins accompanied by synovial hyperplasia and increased synovial fluid, which served as the basis for eligibility for the study and the procedure. There is limited data in the literature regarding the use of embolization in patients with rheumatoid arthritis (RA). Iiboshi et al. present the case of a 79-year-old woman with RA who experienced recurrent joint hemorrhage 11 years after total knee arthroplasty [41]. She underwent successful GAE, resulting in complete resolution of symptoms with no recurrence during a two-year follow-up. An abstract by Badia et al. is available that presents the case of a 40-year-old woman with juvenile idiopathic arthritis who was also successfully treated with GAE [42].

In our study, we used a protocol involving repeated assessments conducted prior to the procedure and at follow-up visits at 1, 3, and 6 months, based on the VAS pain scale ranging from 0 to 10 (on the day of the assessment, the night before, and the average for the week and month preceding the assessment) and the KOOS scale across 5 domains. The range of motion (ROM) and the strength of the extensor (EX) and flexor (FL) muscles of the knee joint on the treated and contralateral limbs were also assessed using specialized electronic devices.

It is believed that GAE reduces excessive vascularization and decreases blood flow through the synovial membrane, thereby alleviating pain associated with inflammation. Blocking areas of hyperemia hinders the arrival of inflammatory mediators, thereby inhibiting further cartilage destruction and synovial neoangiogenesis [43]. In rabbit knee joint models, GAE has been shown to reduce synovial proliferation, hypertrophy, and hyperplasia [44]. Scientific reports indicate that this treatment method is effective in reducing pain and improving patients’ functional status, as reported by the patients themselves. Researchers use several scales to develop a standardized assessment. The most commonly used tools include the VAS for pain assessment (scored on a 0–10 scale or often converted to a 0–100 scale), the WOMAC or KOOS questionnaires, and the Kellgren-Lawrence scale for evaluating changes on radiographs. The WOMAC is a widely used scale consisting of 24 items: 5 on pain, 2 on stiffness, and 17 on physical function. The possible score ranges for each subscale are: 0–20 for pain, 0–8 for stiffness, and 0–68 for physical function [45]. Higher WOMAC scores indicate more severe pain, stiffness, and functional limitations. The KOOS scale consists of 5 sections assessing symptoms and stiffness, pain, daily activities, sports and recreational activities, and quality of life, comprising 42 questions [46]. Each section is assessed separately. The results are converted to a scale from 0 to 100, where 0 indicates severe knee problems and 100 indicates no problems. KOOS is an extension of WOMAC. It has been shown to be a more sensitive and responsive method for younger and more active populations.

An analysis of our results for 34 patients up to 6 months after GAE showed significant improvement in all questionnaire scores, including the VAS and all KOOS subscales. The improvement was noticeable after just 1 month, persisted after 3 months, and remained present after 6 months. The median VAS score on the day of the assessment decreased from 5 [3–6] before the procedure to 2 [1–4] after 1 month, 2 [0–4] after 3 months, and 1.5 [0–4.5] after 6 months (all *p* < 0.001), while the KOOS pain score increased from 64 [47–69] to 81 [72–88.25], 81 [68.5–89], and 83.5 [72.75–92]. Nighttime pain had almost completely subsided. The average VAS score from the week preceding the procedure also decreased from 5 [3.25–7] to 3 [2–4] after 1 month, 3 [2–4] after 3 months, and to 2.5 [1.75–4] after 6 months, and from the month preceding the procedure: 6 [4.25–7.75], 3 [1.75–4.25], 3 [2–4.25], and 3 [2–4.25], respectively. In the other KOOS domains, the following were observed: for symptoms, there was an increase from 55.5 [48–65.25] before the procedure to 79 [69.75–85.5], 80.5 [74.5–89], and 86 [78–89] after treatment at the subsequent time intervals; for ADL, there was an increase from 63 [55.25–80.5] to 85 [74–93], 87 [69–96], and 85 [79–98.5]; for ASR, there was an increase from 25 [10–40] to 50 [40–60.25], 45 [35–54], and 50 [30–73.75]; and for QOL, there was an increase from 38 [28–46.5] pre-procedure to 56 [44–63], 63 [55–64.5], and 53 [46–63.5] post-procedure (all *p* < 0.001).

The findings of other researchers are similar. The greatest reduction in pain is observed one month after the procedure compared to baseline, as measured by both the VAS and the WOMAC and KOOS pain subscales. Over subsequent time periods, pain gradually decreases or remains at a similar level for up to 24 months after GAE. The mean functional scores on the various scales and quality-of-life measures show favorable changes: WOMAC scores tend to decrease and KOOS scores tend to increase, with the highest values observed 1 month after the procedure. They typically decrease or increase gradually depending on the scale, or remain stable in subsequent follow-ups, especially up to 6 months after surgery.

The first report on treatment outcomes by Okuno et al. (2015) described a group of 14 patients using the WOMAC scale [26]. The WOMAC pain score decreased from 12.2 to 3.3 one month after the procedure and to 1.7 after 4 months, while the total WOMAC score decreased from 47.3 to 11.6 one month after the procedure and to 6.3 after 4 months. Another study by Okuno et al., involving 72 patients (95 knees) with KL 1–3 between 2012 and 2016, showed a significant reduction in mean WOMAC pain scores from baseline at various time points following treatment, from 12.1 at baseline to 6.2 at 1 month, 4.4 at 4 months, 3.7 at 6 months, 3.0 at 12 months, and 2.6 at 24 months [38]. The mean total WOMAC score also decreased significantly from 43 to 24, 14.8, 11.2, 8.2, and 6.2 at 1, 4, 6, 12, and 24 months, respectively. The mean VAS score decreased from 72 at baseline to 38, 29, 19, 13, and 14, respectively. A study by Lee et al. in Korea involving 41 patients (71 knees) between 2017 and 2018 showed that mean VAS scores in the group with mild to moderate OA were significantly reduced after 1 day, 1 week, and 1, 3, and 6 months (5.5 at baseline compared with 3.2, 3.1, 2.9, 2.2, and 1.9 after treatment) [47]. A 2020 study by Bagla et al. evaluated 20 patients in the United States, in whom the mean VAS score decreased from 76 at the start of the study to 29 after 6 months of follow-up, and the mean WOMAC score decreased from 61 to 29 after 6 months of follow-up [48].

In the study by Landers et al. involving 10 patients, the median scores for pain, function, and quality of life on the KOOS scale improved by 15.4%, 21.3%, and 100%, respectively, after 12 months [49]. The median KOOS scores were as follows: for KOOS-pain, there was an increase from 54.2 to 70.8 one month after the procedure, 66.7 at 6 months, 62.5 at 12 months, and 51.4 at 24 months; for KOOS-symptoms, there was an increase from 60.7 to 75.0, 64.3, 62.5, and 67.9 in subsequent time intervals; for KOOS-ADL, there was an increase from 65.4 to 80.9, 83.1, 79.4, and 60.3, respectively; for KOOS-ASR, there was an increase from 37.5 to 70.0, 80.0, 45.0, 27.5, and for KOOS-QOL, there was an increase from 25.0 to 59.4, 53.1, 50.0, and 46.9. In 2021, Little et al. evaluated 32 patients with mild to moderate OA who underwent GAE. The mean VAS score in these patients decreased from 60 at the start of the study to 36 after 3 months and 45 after one year [50]. All KOOS subscales showed significant improvement after 6 weeks, 3 months, and 1 year of follow-up, with the exception of activities of daily living: for symptoms, there was an increase from 47.21 to 66.36, 64.40, and 56.92; for pain, there was an increase from 45.46 to 66.40, 62.93, and 57.47; for ADL 52.62 to 70.59, 67.56, and 59.83; for ASR, there was an increase from 20.16 to 39.84, 33.28, and 27.19; for QOL, there was an increase from 21.48 to 46.98, 41.21, and 36.97.

A 2023 study by Gorsi et al. in India involving 10 patients showed that the median VAS score decreased from 7 at the start of the study to 3.5 after 3 months of follow-up, and the median WOMAC score decreased from 53 to 23.50 [51]. In 2024, Hindso et al., in an analysis of the results of a study involving 17 patients with KL 1–3, demonstrated a significant reduction in VAS scores after 6 months compared to baseline values (median of 66 at baseline, 28 after 1 month, 20 after 3 months, and 40 after 6 months) [52]. In the KOOS, pain scores increased from 42 to 72 after 1 month, to 75 after 3 months, and to 64 after 6 months; symptoms scores increased from 50 to 68, 68, and 71, respectively; ADL increased from 46 to 76, 76, and 84; ASR increased from 10 to 50, 60, and 40; and QOL increased from 25 to 50, 50, and 56. A significant decrease in WOMAC subscale scores was also demonstrated.

Joint pain affects isolated motor functions, muscle strength, and movement biomechanics. The quadriceps femoris is the primary muscle responsible for stabilizing the knee joint and absorbing shock while walking. A study by Messier et al. of 480 individuals aged 65 or older with chronic knee pain found a significant decline in balance and lower-limb strength over a 30-month period [53]. Researchers report that knee pain alters neuromuscular activation, often by inhibiting both voluntary and involuntary neural pathways, thereby affecting quadriceps muscle strength (a phenomenon known as arthrogenic muscle inhibition, or AMI) [54]. Persistent neuromuscular changes may alter the response of articular cartilage to joint stress during movement and adversely affect the knee joint [55]. Weakness of the quadriceps muscle associated with muscle dysfunction may be an etiological factor in the development or progression of osteoarthritis [56]. Optimizing the strength of the knee extensor muscles may help prevent knee OA [57]. A systematic review by Culvenor et al. involving over 8000 participants found that knee extensor strength was associated with an increased risk of symptoms and functional decline (WOMAC), but not with an increased risk of radiographic narrowing of the tibiofemoral joint space [58]. Similarly, a meta-analysis by Hou et al. indicated that muscle strength was not significantly associated with an increased risk of radiographic progression in individuals with osteoarthritis or known risk factors for OA [59]. A study by Segal et al. showed that greater quadriceps strength is associated with a reduced risk of symptomatic knee osteoarthritis and a lower risk of progression of tibiofemoral joint space narrowing and cartilage loss in women [60].

Patients diagnosed with knee osteoarthritis have poorer physical fitness and muscle strength compared to the control group, as demonstrated by Liikavainio et al. Passive range of motion of the knee joint, extension strength, and WOMAC scores were associated with disease severity. The extent of radiological changes had a clear negative impact on functional capacity in the later stages of the disease [61]. Diraçoglu’s study found that patients with stage 1 knee osteoarthritis had greater muscle strength than those with stage 2 [62]. The association between low knee extensor strength and worsening tibiofemoral joint OA may be gender dependent, with significant associations observed in women but not in men [63].

The above studies demonstrate the impact of reduced muscle strength on physical fitness, and the range of motion and muscle strength associated with the disease stage may be important when deciding on a course of treatment. A 12-week strength training program combined with a nonsteroidal anti-inflammatory drug (NSAID), glucosamine, or placebo was evaluated in 36 patients with knee osteoarthritis, demonstrating the effect of NSAID or glucosamine administration on muscle strength gains compared to placebo [64]. In a study by Maia et al. of 44 patients treated with viscosupplementation (VS), viscosupplementation with dexamethasone, or dexamethasone, the use of VS alone improved WOMAC scores and knee extensor and flexor strength for up to 6 months after injection [65]. A single intra-articular injection of hyaluronic acid in a study of 30 patients by Tarantino et al. reduced pain and improved joint function after 4 weeks (as measured by VAS and KOOS), without a significant effect on muscle strength on isokinetic testing [66].

In our study of patients after GAE, no significant changes were observed in the parameters of range of motion and strength of the knee extensors and flexors, as well as in the parameters describing the asymmetry between the treated and contralateral limbs in the subsequent time intervals of 1, 3 and 6 months after the procedure. This may be due to the fact that patients did not receive any recommendations for additional exercises to improve muscle mobility and strength after the procedure. In our assessment, the mean knee flexion range in degrees before the procedure was 99.0 [87.75–106.5], 102.0 [95–109.75] after 1 month, 99.45 [95.5–106.5] after 3 months and 104 [93.9–108.75] after 6 months. The mean strength of the knee extensors remained at a similar level over time: 14.05 [11.08–16], 12.0 [10.68–16.72], 11.75 [10.97–15.92], and 11.85 [10.33–13.05]; the flexors showed a similar trend: 7.05 [6–8.47], 7.2 [5.98–8.78], 7.3 [6.75–9], and 7.15 [5.53–8.35], respectively. This means that over the observed period of up to 6 months, a clear clinical improvement was observed by patients, but it was not accompanied by an equally clear improvement in objectively measured functional parameters. However, the analysis of correlations between subjective survey parameters and objective measurements of range of motion and muscle strength showed significant relationships. A greater reduction in pain on the VAS and KOOS-pain within 3 months after the procedure was accompanied by a greater increase in the strength of the extensors and flexors and the range of motion of the limb subjected to the procedure. The reduction in pain and improvement in function were also accompanied by changes in asymmetry between the treated and contralateral sides. After 6 months, subjective improvement, both on the VAS and across all KOOS subscales, was primarily related to an increase in the range of motion of the treated limb, an increase in flexor strength, and changes in asymmetry between the limbs.

Pain weakens the muscles that stabilize the knee joint, disrupts movement biomechanics, and may contribute to the development or progression of osteoarthritis; in turn, degenerative changes and associated symptoms limit movement and functional ability, contributing to a decline in muscle strength. Assuming that the GAE procedure has no direct effect on structural changes but reduces synovial congestion, inflammation, and nociceptors irritation, thereby alleviating disease symptoms and reducing pain, one can expect post-procedure improvement not only in subjective measures but also in objective parameters of movement and muscle strength. Our analysis did not reveal any significant changes in these parameters across successive time intervals. However, a significant correlation was found between increases in muscle strength and range of motion and improvements in subjective parameters. The available literature suggests that pain relief alone may account for improvements in patients’ subjective assessments of their condition, even if biomechanical deficits in the knee joint persist. In line with the concept of so-called arthrogenic muscle inhibition (AMI), pain reduction potentially improves quadriceps activation [54,55]. Once the pain has subsided, the patient finds it easier to move, feels more agile, and rates their function much higher, even though muscle strength, neuromuscular control, or gait pattern may not yet have fully improved. A meta-analysis by Sveaas et al. demonstrated that the use of analgesics alone has a small, but clinically significant effect on improving physical function and walking ability in patients with knee osteoarthritis [67]. The reduction in pain following RFA also significantly improved knee joint function in OA, as demonstrated by Liu et al. in their meta-analysis [68]. It should also be noted that patient satisfaction, expectations, anxiety, and mood significantly influence the assessment of treatment outcomes [69]. A reduction in pain improves a person’s perception of their own physical ability, regardless of their actual level of functionality. The incorporation of psychological interventions into standard care for knee osteoarthritis improves both perceived pain and functional status [70]. It has been suggested that patients who exhibited higher levels of pain catastrophizing at the start of the study (a phenomenon in which a negative assessment of pain intensifies the perception of pain and causes stress) experienced a greater reduction in pain following the GAE procedure [33,36]. In summary, several factors may have contributed to the fact that the reduction in pain following GAE in our study led to functional improvement in patients, even though knee range of motion and muscle strength remained essentially unchanged. It should also be noted that if the results of objective parameters remain unchanged in subsequent time intervals following the procedure, this may indicate that osteoarthritis and structural changes are not progressing. Further long-term observations and research in this area are necessary. Patients who have undergone GAE may experience clinical benefits, potentially delaying or avoiding surgical intervention.

The literature examining the effect of knee artery embolization on the range of motion and muscle strength of the treated and contralateral limbs remain limited. Most studies focus on pain and function assessed by questionnaires rather than direct measures of movement and strength. Functional tests, such as the 30 s chair stand test (30CST) or the 6 min walk test (6MWT), can be indirect measures of lower limb function and strength. In the study by Landers et al., in additional functional tests (30CST and 6MWT), the median results in patients 12 months after GAE surgery improved by 43% and 26%, respectively [49]. Hindsø et al. assessed patients using physical fitness tests at baseline and at 1 and 6 months after embolization: the 40 m brisk walking test, the 30 s chair stand test, and the stair climbing test in 17 patients [52]. Significant improvements were demonstrated in all three tests. The most significant results were for the 30CST, showing a median individual improvement of 45%. However, there is currently no strong evidence that embolization leads to a measurable increase in muscle strength or knee range of motion as assessed by objective biomechanical methods. This is a clear research gap in this field.

Exercise therapy improves range of motion and helps strengthen the muscles of the affected limb, helping prevent muscle weakness that tends to decrease as joint changes progress and pain worsens. Exercises are recommended as part of the treatment for AO to improve pain, stiffness, range of motion, muscle strength, and proprioception [71]. Exercise therapy may help reduce pain, improve functional ability, and enhance quality of life in osteoarthritis, as demonstrated by an analysis by Viderman et al. [72]. Combination therapy involving medication and exercise yields better results than either therapy alone [73]. Implementing exercises after GAE treatment may improve both subjective and objective outcomes. Further research is needed to develop an optimal rehabilitation program, and to conduct long-term follow-up studies to confirm the hypothesis.

In our study, patients undergoing GAE demonstrated Kellgren-Lawrence grade 2 and 3 osteoarthritis (50% each). There was no clear evidence that the degree of radiographic changes was significantly associated with the magnitude of clinical and functional improvement after the procedure.

The KL classification is the most widely used radiological classification system for screening for OA and assessing overall clinical severity, although there is not always agreement on the definition and description of the lesion grades [74]. Most contemporary studies on the efficacy of GAE have focused on patients with early or moderate disease (KL 1–3), but the authors have not clearly demonstrated that the KL stage itself is an independent predictor of improvement in pain and functional outcomes. In their study, Callese et al. analyzed factors associated with clinical success in GAE, defined as ≥ 50% improvement on the WOMAC scale 1 year after the procedure. Among 236 patients undergoing GAE between May 2018 and September 2022 (KL grade 1—10 patients; 2—58; 3—96; 4—70), overall clinical success was recorded in 128 patients (54.2%), particularly in younger patients with a higher BMI [75]. Regarding KL grade, clinical success was observed in 7 (5.5%) patients with grade 1, 38 (29.9%) with grade 2, 55 (43.3%) with grade 3 and 27 (21.3%) with grade 4. Factors correlating with clinical success included: BMI, K-L stage, and sparing of the descending geniculate artery. In the study by Mabud et al. analyzing predictors of response to GAE treatment, it was shown that a lower degree of KL changes on the contralateral side of the untreated knee was associated with better WOMAC pain scores after GAE [76]. This raises the possibility that less severe disease on the contralateral side may reduce the compensatory biomechanical loading in the treated knee. On the other hand, patients with advanced contralateral knee OA may experience changes in gait mechanics and increased weight bearing, potentially limiting symptom improvement following GAE. Most available data indicate that the best results are achieved in patients with KL 1–3, and the worst in patients with KL 4. However, this relationship has not been confirmed; therefore the degree of KL degenerative changes should be considered as a prognostic factor in the treatment of GAE.

In the same study by Callese et al. described above, no significant difference was observed in the total number of embolized arteries [75]. Also, in our study, the number of embolized vessels was not significantly associated with the magnitude of clinical and functional improvement after the procedure. In the available publications, the influence of the number of vessels simultaneously embolized as an independent factor on the clinical outcome is poorly investigated. Most authors report the number of embolized branches but do not formally analyze the relationship between the number of vessels and treatment efficacy. This is one of the research gaps in this field.

GAE has a favorable safety profile. Most adverse events are mild and transient, consisting mainly of post-procedural pain that resolves on its own, low-grade fever, temporary skin changes (erythema or discoloration), and paresthesia in the foot. Serious complications are rare but may include skin ischemia and asymptomatic bone changes [5,21,33,36]. Our study did not reveal any serious complications. Large hematomas were observed in two patients (5.9%), which resolved spontaneously. It is essential to standardize surgical procedures for GAE and postoperative care to avoid potential complications. The introduction of innovative technologies into knee surgery requires a thorough safety assessment and standardized reporting of outcomes and adverse events [77].

It is also important to note the suggested contraindications for the procedure, including severe peripheral arterial disease (due to the risk of impaired collateral circulation in the lower extremities), active infection of the knee joint, allergy to contrast agents, and renal failure [36]. Autoimmune and inflammatory diseases are also listed among the potential contraindications for GAE, including those caused by other sources of pain. Nevertheless, after a thorough analysis of the imaging studies and the patient’s condition, the procedure may be considered. Preliminary results from our study of patients diagnosed with RA, in whom active inflammation was ruled out and imaging of the knee joint revealed chondropathic and degenerative changes with synovial hypertrophy and hyperemia, are encouraging. Further observation and research are necessary.

Several limitations of this study should be acknowledged. This study was a single-center prospective study. The absence of a control group significantly limits the interpretation of the results. Relatively small sample cannot present all aspects of the natural course of the disease. The inclusion of two patients with rheumatoid arthritis-related synovitis represents a potential source of clinical heterogeneity. However, because rheumatoid arthritis accounted for only two cases, a formal subgroup analysis was not statistically justified. The OA-only sensitivity analysis confirmed that excluding these patients did not materially change the main conclusions. The present analysis is focused mainly on short-term outcomes up to 6 months. Further observation is necessary to assess the long-term effectiveness of GAE. The objective functional outcomes should be interpretated cautiously because the study was powered mainly to detect moderate or larger within-subject effects. Therefore, smaller changes in ROM or muscle strength may have remained undetected. Large cohorts and long-term studies comparing GAE with standard conservative treatment, sham procedures, and other treatments for chronic synovitis and osteoarthritis of the knee are needed to determine the extent of the therapeutic effect attributable to embolization. Innovative digital monitoring systems that enable the maintenance of multicenter registries and the secure collection of medical records and long-term outcomes can improve the monitoring of innovative procedures [78].

Despite these limitations, the prospective design, repeated assessments and high follow-up completeness represent important strengths of the present study.

## 5. Conclusions

At the 6-month follow-up after knee artery embolization, patients with exudative synovitis of the knee joint achieved significant clinical improvement in perceived pain, symptoms, daily activities, sports and recreation, and quality of life. However, no clear improvement was observed in other objective parameters, such as range of motion or muscle strength of the knee joint. While the early outcomes of the procedure are encouraging, they need to be confirmed by long-term follow-up.

GAE shows promise as an adjunctive treatment for exudative synovitis associated with osteoarthritis, particularly in patients for whom conservative treatment is insufficient but who do not yet require surgery. Randomized clinical trials with adequate statistical power and long-term follow-up are needed to determine the true clinical value of GAE and to define its optimal role in the treatment algorithm for knee osteoarthritis.

## Figures and Tables

**Table 1:** Completeness of follow-up in the main cohort.

Study Number	Time Point	*n*/*N*	%
Study 1 (B1)	the day before/on the day of the procedure	34/34	100.0
Study 2 (B2)	1 month after the procedure	34/34	100.0
Study 3 (B3)	3 months after the procedure	32/34	94.1
Study 4 (B4)	6 months after the procedure	26/34	76.5
Study 5 (B5)	12 months after the procedure	8/34	23.5

**Table 2:** Characteristics of the study cohort.

Feature	Result
Main cohort	34 episodes
Women	17 (50.0%)
Men	17 (50.0%)
Age [years]	60.0 [52.2–63.8]
BMI [kg/m^2^]	32.0 [28.5–35.6]
Duration of symptoms [years]	1.75 [0.62–2.50]
Right knee	18 (52.9%)
Left knee	16 (47.1%)
OA	32 (94.1%)
RA	2 (5.9%)
Grade 2 KL	17 (50.0%)
Grade 3 KL	17 (50.0%)
Healthy weight	6 (17.6%)
Overweight	7 (20.6%)
Class I Obesity	11 (32.4%)
Class II Obesity	8 (23.5%)
Class III Obesity	2 (5.9%)
HT	14 (14.2%)
IHD	3 (8.8%)
T2DM	2 (5.9%)
AF	2 (5.9%)
NSAIDs	34 (100.0%)
Opioids	4 (11.8%)
Oral GCSs	3 (8.8%)
MTX	2 (5.9%)
Number of intra-articular injections	3 [3.0–4.0]
GCS	15 (44.1%)
HA	31 (91.2%)
PRP	22 (64.7%)
Number of embolized vessels	3 [3.0–4.0]
Complications	2 (5.9%)—extensive hematoma
Post-procedural rehabilitation	5 (14.7%)
Additional intra-articular injections	3 (8.8%)
Further surgery or re-embolization	3 (8.8%)

BMI—body mass index; KL—Kellgren-Lawrence; OA—osteoarthritis; RA—rheumatoid arthritis; HT—hypertension; IHD—ischemic heart disease; T2DM—type 2 diabetes mellitus; AF—atrial fibrillation; NSAIDs—Nonsteroidal anti-inflammatory drugs; GCS—glucocorticosteroid; MTX—methotrexate; HA—hyaluronic acid; PRP—Platelet-rich plasma. Data are presented as median [IQR] or *n* (%).

**Table 3:** Changes in clinical and functional outcomes at 6 months.

Variable with Model-Based Estimate (95% CI) and d	Study				*p* Time Effect
	Before the Procedure	1 Month After the Procedure	3 Months After the Procedure	6 Months After the Procedure	
A. VAS					
Day of assessment	5 [4–6]	2 [1–3] ***	2 [0.75–3.25] ***	1.5 [1–2.25] ***	<0.001
		−2.15 (−3.01;−1.28) d = −1.47	−2.52 (−3.41;−1.64) d = −1.72	−2.71 (−3.65;−1.76) d = −1.85	
Night before assessment	1.5 [1–2.75]	0 [0–1] ***	0 [0–1] **	0 [0–1] **	<0.001
		−1.76 (−2.83;−0.70) d = −0.98	−1.53 (−2.61;−0.44) d = −0.85	−1.61 (−2.77;−0.45) d = −0.89	
Weekly average	5 [3.25–7]	3 [2–4] ***	3 [2–4] ***	2.5 [1.75–4] ***	<0.001
		−2.09 (−3.20;−0.98) d = −1.11	−2.26 (−3.39;−1.13) d = −1.20	−2.45 (−3.66;−1.25) d = −1.31	
Monthly average	6 [4.25–7.75]	3 [1.75–4.25] ***	3 [2–4.25] ***	3 [2–4.25] ***	<0.001
		−2.00 (−2.95;−1.05) d = −1.24	−2.93 (−3.90;−1.96) d = −1.82	−2.86 (−3.90;−1.82) d = −1.77	
B. KOOS subscales					
Symptoms	55.5 [48–65.25]	79 [69.75–85.5] ***	80.5 [74.5–89] ***	86 [78–89] ***	<0.001
		21.03 (13.42;28.64) d = 1.63	22.61 (14.84;30.37) d = 1.76	24.37 (16.05;32.70) d = 1.89	
Pain	64 [56–69]	81 [75–88.25] ***	81 [74.5–89] ***	83.5 [76.5–90] ***	<0.001
		18.03 (11.46;24.60) d = 1.62	17.00 (10.30;23.70) d = 1.53	19.86 (12.68;27.04) d = 1.79	
ADL	63 [55.25–80.5]	85 [74–93] ***	87 [69–96] ***	85 [79–98.5] ***	<0.001
		17.94 (11.98;23.90) d = 1.78	18.84 (12.75;24.93) d = 1.87	19.63 (13.10;26.16) d = 1.95	
ASR	25 [10–40]	50 [40–60.25] ***	45 [35–54] ***	50 [30–73.75] ***	<0.001
		23.24 (18.86;31.61) d = 1.64	23.03 (14.47;31.58) d = 1.63	26.99 (17.81;36.18) d = 1.91	
QOL	38 [28–46.5]	56 [44–63] ***	63 [55–64.5] ***	53 [46–63.5] ***	<0.001
		18.56 (11.74;25.37) d = 1.61	19.67 (12.71;26.62) d = 1.71	20.91 (13.47;28.36) d = 1.81	
C. Range of motion and muscle strength					
ROM	99.0 [87.75–106.5]	102.0 [95–109.75]	99.45 [95.5–106.5]	104 [93.9–108.75]	0.110
		2.83 (−1.24;6.90) d = 0.33	4.84 (2.34;8.02) d = 0.56	4.20 (−1.36;9.76) d = 0.49	
EX	14.05 [11.08–16]	12.0 [10.68–16.72]	11.75 [10.97–15.92]	11.85 [10.33–13.05]	0.204
		0.90 (−1.61;3.41) d = 0.21	−0.51 (−3.06;2.05) d = −0.12	−1.41 (−4.15;1.32) d = −0.33	
FL	7.05 [6–8.47]	7.2 [5.98–8.78]	7.3 [6.75–9]	7.15 [5.53–8.35]	0.616
		0.19 (−0.90;1.27) d = 0.10	0.15 (−0.96;1.26) d = 0.08	−0.40 (−1.59;0.78) d = −0.22	
D. Asymmetry					
ΔROM	−6.6 [−14.95–−1]	−5.85 [−9–4]	−6.6 [−10–2]	−3.7 [−11.23–3.4]	0.331
		3.70 (−1.54;8.95) d = 0.42	1.43 (−3.92;6.78) d = 0.16	2.88 (−2.85;8.60) d = 0.32	
ΔEX	−0.2 [−2.05–0.67]	−1.35 [−2.53–1.62]	−0.70 [−1.2–1.45]	−0.05 [−1.17–0.72]	0.230
		−0.54 (−2.3;1.04) d = −0.20	0.42 (−1.19;2.03) d = 0.16	0.81 (−0.90;2.51) d = 0.30	
ΔFL	−0.7 [−1.82–0.02]	−0.6 [−1.55–1.1]	−0.1 [−1.75–0.87]	−0.25 [−1.17–0.725]	0.368
		0.29 (−0.52;1.09) d = 0.21	0.57 (−0.25;1.39) d = 0.42	0.43 (−0.45;1.31) d = 0.31	

ADL—activities of daily living; ASR—sports and recreational activities; QOL—quality of life; ROM—range of motion; EX—extensor muscle strength; FL—flexor muscle strength; ΔROM—difference in range of motion between the treated limb and the contralateral limb; ΔEX—difference in extensor strength between the treated limb and the contralateral limb; ΔFL—difference in flexor strength between the treated limb and the contralateral limb. Data are presented as median [IQR]. Model-based changes are presented as estimated differences from baseline with 95% confidence intervals. Standardized effect size d was calculated as the model-based contrast estimate divided by the residual standard deviation of the corresponding linear mixed model. For the VAS, negative model-based changes and negative d values indicate pain reduction. For descriptive interpretation, the absolute values of standardized effect sizes were considered negligible when |d| < 0.2, small around 0.2, moderate around 0.5, and large when |d| ≥ 0.8. Asterisks indicate the significance of planned comparisons relative to the baseline value (B1) in mixed models: ** *p* < 0.01; *** *p* < 0.001 (Holm correction). The last column presents the *p*-value for the global effect of time.

**Table 4:** Sensitivity power analysis for objective functional parameters.

Objective Parameter	Follow-Up Comparison	Available Paired n	Minimum Detectable Standardized Effect	Minimum Detectable Change in Original Units
ROM	B2 vs. B1	34	0.50	7.12°
	B3 vs. B1	32	0.51	7.63°
	B4 vs. B1	26	0.57	7.21°
EX	B2 vs. B1	34	0.50	3.59 kg
	B3 vs. B1	32	0.51	2.93 kg
	B4 vs. B1	26	0.57	2.81 kg
FL	B2 vs. B1	34	0.50	1.47 kg
	B3 vs. B1	32	0.51	1.29 kg
	B4 vs. B1	26	0.57	1.38 kg

ROM—range of motion; EX—extensor muscle strength; FL—flexor muscle strength. B1—baseline; B2—1 month; B3—3 months; B4—6 months. Minimum detectable effects were estimated for 80% power and a two-sided alpha level of 0.05. Minimum detectable changes in original units were calculated using the observed standard deviation of within-subject changes.

**Table 5:** Comparison of the magnitude of changes between patients with KL 2 and KL 3 at 3 and 6 months post-embolization.

Parameter	3 Months			6 Months		
	KL 2	KL 3	*p*	KL 2	KL 3	*p*
dVAS—day of assessment	3 [1–4]	2.5 [1–3]	0.340	3 [1.5–4]	2 [2–3]	0.671
dVAS—night before	2.5 [0–6]	0 [0–0.5]	0.058	1 [0–4.5]	0 [0–2]	0.417
dVAS—weekly average	2.5 [1–4.25]	2 [0.5–4]	0.447	3 [1–5]	2 [0.5–3]	0.617
dVAS—monthly average	3.5 [1.75–5]	2 [1–4.25]	0.518	3 [1.5–5]	1 [0.5–4.5]	0.432
dKOOS—symptoms	18 [13.25–27.5]	19.5 [6.25–36.75]	0.880	18 [12–36]	22 [2–37.5]	0.897
dKOOS—pain	17 [6.5–36.75]	14 [2.25–21.25]	0.299	25 [2.5–42]	17 [3–26]	0.311
dKOOS—ADL	15.5 [12.5–34]	11.5 [6–21.25]	0.157	15 [8.5–37]	18 [4.5–23.5]	0.483
dKOOS—ASR	30 [17.5–40]	20 [3.75–31.25]	0.198	25 [10–45]	25 [5–40]	0.514
dKOOS—QOL	19 [16.75–31]	12.5 [−6.25–31.25]	0.198	25 [7–47]	13 [3–28]	0.131
dROM	5.65 [−5.65–17.25]	0.75 [−3.2–5.88]	0.474	7.9 [1.85–10.95]	1 [−5.25–8.35]	0.406
dEX	0.55 [−3.05–4.23]	−1.55 [−4.88–1.3]	0.250	−2.3 [−4.5–0.65]	−2 [−4.75–0.9]	0.959
dFL	0.6 [−1.58–2.12]	0.45 [−1.32–1.27]	0.585	−0.9 [−1.9–1.4]	0 [−1.1–1.5]	0.640
dΔROM	3.1 [−5.47–15.55]	−3.4 [−8–3.9]	0.158	11 [0.8–13.45]	−1 [−6.25–8.5]	0.194
dΔEX	−0.75 [−2.1–2.9]	0.35 [−2.93–3.22]	0.955	0.3 [−1.55–1]	0 [−1.25–2.5]	0.678
dΔFL	1.15 [−1.03–1.52]	1.15 [−0.03–1.8]	0.462	0.2 [−0.4–2]	0.7 [−0.9–2.65]	0.604

Data are presented as median [IQR]. *p*-values were obtained using the Mann–Whitney U test to compare the magnitude of changes between patients with KL 2 and KL 3. d—the change in the parameter relative to baseline study. KL—Kellgren-Lawrence; ADL—activities of daily living; ASR—sports and recreational activities; QOL—quality of life; ROM—range of motion; EX—extensor strength; FL—flexor strength.

**Table 6:** Spearman’s correlations between the number of embolized vessels and the magnitude of changes in parameters 3 and 6 months after the procedure.

Parameter	3 Months		6 Months	
	r_S_	*p*	r_S_	*p*
dVAS—day of assessment	−0.113	0.539	−0.180	0.379
dVAS—night before	0.019	0.917	−0.141	0.492
dVAS—weekly average	−0.014	0.941	−0.031	0.881
dVAS—monthly average	0.025	0.892	0.011	0.956
dKOOS—symptoms	−0.118	0.521	−0.082	0.691
dKOOS—pain	0.089	0.630	0.069	0.739
dKOOS—ADL	0.087	0.634	0.016	0.939
dKOOS—ASR	0.134	0.465	0.135	0.510
dKOOS—QOL	0.045	0.808	0.080	0.698
dROM	−0.045	0.805	0.051	0.806
dEX	0.019	0.920	0.220	0.281
dFL	−0.101	0.584	0.049	0.812
dΔROM	0.168	0.357	0.261	0.198
dΔEX	0.364	0.041	0.248	0.221
dΔFL	0.109	0.554	0.046	0.825

The rs coefficients and *p*-values were obtained using Spearman’s rank correlation analysis. d—the change in the parameter relative to the baseline study. ADL—activities of daily living; ASR—sports and recreational activities; QOL—quality of life; ROM—range of motion; EX—extensor strength; FL—flexor strength.

**Table 7:** Spearman’s correlations between changes in subjective and objective parameters—3 months.

	d VAS				d KOOS				
Parameter	Day of Assessment	Night Before	Weekly Average	Monthly Average	Symptoms	Pain	ADL	ASR	QOL
dROM	r_S_ = 0.203 *p* = 0.265	r_S_ = 0.149 *p* = 0.415	r_S_ = 0.370 *p* = 0.037	r_S_ = 0.191 *p* = 0.295	r_S_ = 0.217 *p* = 0.233	r_S_ = 0.367 *p* = 0.039	r_S_ = 0.349 *p* = 0.050	r_S_ = 0.213 *p* = 0.242	r_S_ = 0.408 *p* = 0.020
dEX	r_S_ = 0.497 *p* = 0.004	r_S_ = 0.419 *p* = 0.017	r_S_ = 0.402 *p* = 0.023	r_S_ = 0.208 *p* = 0.254	r_S_ = 0.399 *p* = 0.024	r_S_ = 0.555 *p* < 0.001	r_S_ = 0.411 *p* = 0.020	r_S_ = 0.292 *p* = 0.104	r_S_ = 0.348 *p* = 0.051
dFL	r_S_ = 0.175 *p* = 0.337	r_S_ = 0.143 *p* = 0.433	r_S_ = 0.234 *p* = 0.197	r_S_ = 0.131 *p* = 0.475	r_S_ = 0.346 *p* = 0.052	r_S_ = 0.430 *p* = 0.014	r_S_ = 0.332 *p* = 0.063	r_S_ = 0.156 *p* = 0.395	r_S_ = 0.222 *p* = 0.221
dΔROM	r_S_ = 0.155 *p* = 0.396	r_S_ = 0.206 *p* = 0.259	r_S_ = 0.386 *p* = 0.029	r_S_ = 0.210 *p* = 0.248	r_S_ = 0.248 *p* = 0.171	r_S_ = 0.427 *p* = 0.015	r_S_ = 0.372 *p* = 0.036	r_S_ = 0.183 *p* = 0.315	r_S_ = 0.534 *p* = 0.002
dΔEX	r_S_ = 0.138 *p* = 0.451	r_S_ = 0.204 *p* = 0.262	r_S_ = 0.399 *p* = 0.024	r_S_ = 0.211 *p* = 0.247	r_S_ = 0.197 *p* = 0.280	r_S_ = 0.286 *p* = 0.113	r_S_ = 0.269 *p* = 0.137	r_S_ = 0.252 *p* = 0.163	r_S_ = 0.210 *p* = 0.248
dΔFL	r_S_ = 0.083 *p* = 0.653	r_S_ = 0.233 *p* = 0.200	r_S_ = 0.121 *p* = 0.510	r_S_ = 0.127 *p* = 0.487	r_S_ = 0.201 *p* = 0.271	r_S_ = 0.369 *p* = 0.038	r_S_ = 0.024 *p* = 0.898	r_S_ = −0.034 *p* = 0.852	r_S_ = 0.224 *p* = 0.218

d—change in the parameter at 3 months compared to the pre-study assessment; ROM—range of motion; EX—extensor strength; FL—flexor strength; ΔROM, ΔEX, and ΔFL—change in asymmetry between the treated limb and the contralateral limb in range of motion, extensor strength, and flexor strength, respectively; ADL—activities of daily living; ASR—sports and recreational activities; QOL—quality of life.

**Table 8:** Spearman’s correlations between changes in subjective and objective parameters—6 months.

	d VAS				d KOOS				
Parameter	Day of Assessment	Night Before	Weekly Average	Monthly Average	Symptoms	Pain	ADL	ASR	QOL
dROM	r_S_ = 0.425 *p* = 0.031	r_S_ = 0.437 *p* = 0.026	r_S_ = 0.615 *p* = <0.001	r_S_ = 0.301 *p* = 0.136	r_S_ = 0.561 *p* = 0.003	r_S_ = 0.664 *p* < 0.001	r_S_ = 0.512 *p* = 0.008	r_S_ = 0.459 *p* = 0.018	r_S_ = 0.431 *p* = 0.028
dEX	r_S_ = 0.043 *p* = 0.833	r_S_ = 0.095 *p* = 0.643	r_S_ = −0.008 *p* = 0.969	r_S_ = −0.149 *p* = 0.468	r_S_ = −0.023 *p* = 0.911	r_S_ = −0.160 *p* = 0.435	r_S_ = −0.030 *p* = 0.885	r_S_ = −0.171 *p* = 0.404	r_S_ = −0.101 *p* = 0.624
dFL	r_S_ = 0.302 *p* = 0.133	r_S_ = 0.283 *p* = 0.162	r_S_ = 0.454 *p* = 0.020	r_S_ = 0.267 *p* = 0.187	r_S_ = 0.433 *p* = 0.027	r_S_ = 0.528 *p* = 0.006	r_S_ = 0.354 *p* = 0.076	r_S_ = 0.444 *p* = 0.023	r_S_ = 0.483 *p* = 0.012
dΔROM	r_S_ = 0.212 *p* = 0.298	r_S_ = 0.283 *p* = 0.161	r_S_ = 0.469 *p* = 0.016	r_S_ = 0.216 *p* = 0.290	r_S_ = 0.429 *p* = 0.029	r_S_ = 0.489 *p* = 0.011	r_S_ = 0.398 *p* = 0.044	r_S_ = 0.241 *p* = 0.235	r_S_ = 0.411 *p* = 0.037
dΔEX	r_S_ = −0.318 *p* = 0.114	r_S_ = 0.050 *p* = 0.807	r_S_ = −0.380 *p* = 0.056	r_S_ = −0.431 *p* = 0.028	r_S_ = −0.192 *p* = 0.349	r_S_ = −0.139 *p* = 0.497	r_S_ = −0.069 *p* = 0.739	r_S_ = −0.200 *p* = 0.326	r_S_ = −0.214 *p* = 0.293
dΔFL	r_S_ = 0.147 *p* = 0.472	r_S_ = 0.249 *p* = 0.220	r_S_ = 0.281 *p* = 0.165	r_S_ = 0.255 *p* = 0.209	r_S_ = 0.401 *p* = 0.043	r_S_ = 0.369 *p* = 0.063	r_S_ = 0.216 *p* = 0.290	r_S_ = 0.467 *p* = 0.016	r_S_ = 0.293 *p* = 0.147

d—change in the parameter at 6 months compared to the pre-study assessment; ROM—range of motion; EX—extensor strength; FL—flexor strength; ΔROM, ΔEX, and ΔFL—change in asymmetry between the treated limb and the contralateral limb in range of motion, extensor strength, and flexor strength, respectively; ADL—activities of daily living; ASR—sports and recreational activities; QOL—quality of life.

## Data Availability

Data is contained within the article.

## Abbreviations

The following abbreviations are used in this manuscript:

OA	Osteoarthritis
PRP	Platelet-rich plasma
GCSs	Glucocorticosteroids
HA	Hyaluronic acid
BMAC	Bone marrow aspirate concentrate
RFA	Radiofrequency ablation
HT	Hypertension
IHD	Ischemic heart disease
AF	Atrial fibrillation
T2DM	Type 2 diabetes mellitus
GAE	Genicular artery embolization
NSAIDs	Nonsteroidal anti-inflammatory drugs
No.	Number
KL	Kellgren-Lawrence
BMI	Body Mass Index
VAS	Visual Analog Scale
KOOS	Knee Injury and Osteoarthritis Outcome Study
ADL	Activities of daily living
ASR	Sports and recreational activities
QOL	Quality of life
ROM	Range of motion
EX	Extensor
FL	Flexor
IQR	Interquartile range
CI	Confidence interval
LMM	Linear mixed model
RA	Rheumatoid arthritis
VS	Viscosupplementation
AMI	Arthrogenic muscle inhibition
30 CST	30 s chair stand test
6MWT	6 min walk test
vs.	versus

## Appendix A

### Appendix A.1

Table A1 presents a comparison of the results of the main and sensitivity analyses after excluding observations obtained after additional post-procedural interventions. For each variable, the significance of the global time effect and planned comparisons with the pre-procedure assessment are reported.

**Table A1:** OA-only sensitivity analysis after excluding rheumatoid arthritis cases.

Variable	Time Effect		B2 vs. B1		B3 vs. B1		B4 vs. B1		Interpretation
	Main Analysis	Sensitivity Analysis	Main Analysis	Sensitivity Analysis	Main Analysis	Sensitivity Analysis	Main Analysis	Sensitivity Analysis	
VAS									
day of assessment	<0.001	<0.001	<0.001	<0.001	<0.001	<0.001	<0001	<0.001	significant time effect
Night before assessment	<0.001	<0.001	<0.001	<0.001	0.002	<0.001	0.002	<0.001	significant time effect
weekly average	<0.001	<0.001	<0.001	<0.001	<0.001	<0.001	<0.001	<0.001	significant time effect
monthly average	<0.001	<0.001	<0.001	<0.001	<0.001	<0.001	<0.001	<0.001	significant time effect
KOOS									
symptoms	<0.001	<0.001	<0.001	<0.001	<0.001	<0.001	<0.001	<0.001	significant time effect
pain	<0.001	<0.001	<0.001	<0.001	<0.001	<0.001	<0.001	<0.001	significant time effect
ADL	<0.001	<0.001	<0.001	<0.001	<0.001	<0.001	<0.001	<0.001	significant time effect
ASR	<0.001	<0.001	<0.001	<0.001	<0.001	<0.001	<0.001	<0.001	significant time effect
QOL	<0.001	<0.001	<0.001	<0.001	<0.001	<0.001	<0.001	<0.001	significant time effect
Objective									
ROM of treated limb	0.110	0.113	0.177	0.137	0.075	0.087	0.137	0.137	no significant time effect
EX of treated limb	0.204	0.198	0.769	0.711	0.769	0.711	0.631	0.711	no significant time effect
FL of treated limb	0.616	0.728	1.000	1.000	1.000	1.000	1.000	1.000	no significant time effect
Asymmetry									
ΔROM	0.331	0.158	0.265	0.075	0.516	0.410	0.448	0.410	no significant time effect
ΔEX	0.230	0.389	0.815	1.000	0.815	1.000	0.752	0.812	no significant time effect
ΔFL	0.368	0.327	0.474	0.584	0.283	0.237	0.474	0.529	no significant time effect

B1—pre-procedure/on the day of the procedure assessment; B2—1-month follow-up; B3—3-month follow-up; B4—6-month follow-up; ROM—range of motion; EX—extensor strength; FL—flexor strength; ΔROM, ΔEX, and ΔFL—differences in asymmetry between the treated limb and the contralateral limb in range of motion, extensor strength, and flexor strength, respectively.

### Appendix A.2

Table A2 presents a comparison of the results of the main analysis and the sensitivity analysis after excluding observations obtained following additional post-procedure interventions. For each variable, the significance of the overall effect of time and the planned comparisons relative to the pre-procedure assessment is summarized.

**Table A2:** Comparison of the results of the main analysis and the sensitivity analysis.

Variable	Time Effect		B2 vs. B1		B3 vs. B1		B4 vs. B1		Conclusion
	Main Analysis	Sensitivity Analysis	Main Analysis	Sensitivity Analysis	Main Analysis	Sensitivity Analysis	Main Analysis	Sensitivity Analysis	
VAS									
day of assessment	<0.001	<0.001	<0.001	<0.001	<0.001	<0.001	<0001	<0.001	stable result
night before	<0.001	<0.001	<0.001	<0.001	0.002	<0.001	0.002	<0.001	stable result
weekly average	<0.001	<0.001	<0.001	<0.001	<0.001	<0.001	<0.001	<0.001	stable result
monthly average	<0.001	<0.001	<0.001	<0.001	<0.001	<0.001	<0.001	<0.001	stable result
KOOS									
symptoms	<0.001	<0.001	<0.001	<0.001	<0.001	<0.001	<0.001	<0.001	stable result
pain	<0.001	<0.001	<0.001	<0.001	<0.001	<0.001	<0.001	<0.001	stable result
ADL	<0.001	<0.001	<0.001	<0.001	<0.001	<0.001	<0.001	<0.001	stable result
ASR	<0.001	<0.001	<0.001	<0.001	<0.001	<0.001	<0.001	<0.001	stable result
QOL	<0.001	<0.001	<0.001	<0.001	<0.001	<0.001	<0.001	<0.001	stable result
Objective									
ROM	0.110	0.074	0.177	0.203	0.075	0.052	0.137	0.119	stable result
EX	0.204	0.297	0.769	1.000	0.769	1.000	0.631	0.447	stable result
FL	0.616	0.749	1.000	1.000	1.000	1.000	1.000	1.000	stable result
Asymmetry									
ΔROM	0.331	0.186	0.265	0.157	0.516	0.238	0.448	0.202	stable result
ΔEX	0.230	0.206	0.815	0.781	0.815	0.728	0.752	0.781	stable result
ΔFL	0.368	0.368	0.474	0.679	0.283	0.265	0.474	0.679	stable result

B1—study the day before/on the day of the procedure; B2—1 month after the procedure; B3—3 months after the procedure; B4—6 months after the procedure; ROM—range of motion; EX—extensor strength; FL—flexor strength; ΔROM, ΔEX, and ΔFL—change in asymmetry between the operated limb and the contralateral limb in range of motion, extensor strength, and flexor strength, respectively.
